# Mesenchymal Stem Cell-Based Therapy for Allergic Rhinitis

**DOI:** 10.1155/2020/2367524

**Published:** 2020-06-10

**Authors:** Liwei Sun, Jichao Sha, Cuida Meng, Dongdong Zhu

**Affiliations:** Department of Otolaryngology Head and Neck Surgery, China-Japan Union Hospital of Jilin University, Changchun, China

## Abstract

Allergic rhinitis (AR) is a prevalent disorder that causes a significant and often underestimated health burden for individuals and society. The current drug treatment cannot essentially deal with the regulation of the allergic reaction, while the allergic symptoms could be alleviated. Mesenchymal stem cells (MSCs) bear a variety of properties, such as the ability to differentiate into various cell lineages, to secrete soluble factors crucial for cell survival and proliferation, to migrate to the exact site of injury, and to modulate the immune response. Clinical studies have been extensively conducted in MSCs as the models for varieties of diseases such as neurological diseases. Due to their immunomodulatory properties, the MSCs have gradually been believed to become one of the promising strategies for AR treatments although so far the MSCs-mediated treatment for AR is still at animal experiments stage. Fully understanding the roles and mechanisms of MSCs immunomodulatory effects serves as the prerequisite that will be beneficial to the application of MSCs-based AR clinical treatment methods. In this review article, we highlighted the recent research advances and give a brief perspective in the future study of the MSCs-mediated therapeutic application in AR treatments.

## 1. Introduction

Characterized by the presence of one or more nasal symptoms, including sneezing, itching, nasal discharge, and nasal congestion, allergic rhinitis (AR) has been identified as a noninfectious chronic inflammatory disease of the nasal mucosa. Pathologically, the AR is associated with immunoglobulin E (IgE)-mediated immune responses against environmental allergens [1]. The epidemiological studies show that the prevalence of AR is gradually increasing in more developed countries, currently affecting 10%-40% of adults and 2%-25% of children worldwide [2–5]. Atopy is characterized by the production of allergen-specific IgE against environmental allergens. Atopy individuals are sensitive to allergens via activating dendritic cells (DCs) and T lymphocytes (T cells). It is well known that the DCs are located on the surface of the nasal mucosa capture allergens and could present allergen peptides to T cells in the draining lymph nodes to cause a T-helper 2(Th2)-type allergic reaction. Consequently, the release of Th2-related cytokines enhances the IgE production by B-lymphocytes (B cells) and promotes the recruitment of eosinophils in nasal tissue. More specifically, the IgE molecules are released into the blood and bind to high-affinity receptors on the surface of tissue mast cells and circulating basophils. Pathophysiologically, allergens bind to allergen-specific IgE on the surface of mast cells, leading to the rapid release of preformed mediators (such as histamine) and consequently causing early symptoms such as sneezing, nasal itching, and rhinorrhoea. Histamine and tumor necrosis factor-*α*(TNF-*α*), as well as newly generated lipid mediators such as leukotriene C4 and prostaglandin D2, all contribute to the influx of inflammatory cells such as eosinophils, basophils, and CD4+ T cells by stimulating the expression of adhesion molecules on endothelial cells, causing late symptom such as nasal congestion [6–8]. At present, regular drug treatment could alleviate the allergic symptoms, but could not interfere the allergic reactions. The recurrence of symptoms and side effects of the drugs applied for treatments confer the significant drug resistance to the patients, severely affecting patients' quality of life. On the other hand, however, this situation inspires the related medical scientists to look for more effective strategies for AR treatments.

MSCs are identified to be pluripotent, nonhematopoietic, stromal precursor cells in adult, and neonatal tissues. The most common sources of MSCs are bone marrow, adipose tissue, and umbilical cord [9]. Bearing the potentiality for self-renewal and multidirectional differentiation, the MSCs are thought to function as tissue repair and increasingly believed to be regulators of the immune response. Given their immunosuppressive properties, tissue repair capacity, and secretion of various biological factors, the MSCs are being considered as a promisingly potential source for the AR treatment. The clinical study has been conducted for a variety of diseases, including cardiovascular diseases, neurological diseases, bone and cartilage disease, liver, lung, and kidney injury, organ transplantation, chronic inflammatory, and autoimmune diseases [10]. However, long way is expected to go for the clinical study in AR patients. In this review, the current status of MSCs in AR treatments was highlighted particularly the immunomodulatory properties of MSCs and their therapeutic potential in animal models of AR. As a perspective, we discuss the study directions in the future as well as the challenges to be overcome for the MSCs-based clinical AR therapy.

## 2. Overview of the Current Therapeutic Strategies

Generally speaking, the current approaches for the AR therapy include prevention of allergen or irritant contact, pharmacotherapy, specific immunotherapy, and surgery. However, almost all these strategies are symptoms—alleviating based passive approaches. Whether selected by patients themselves or prescribed by medical personnel, pharmacotherapy serves as the main approach to control the symptoms of AR. There are numerous options for oral or systemic use, topical intranasal application, and alternative therapies that can be considered. Pharmacotherapy includes mast cell stabilizers, antihistamines, glucocorticosteroids (GCSs), leukotriene receptor antagonists, and nasal decongestants [11]. The AR pharmacotherapy could simply control the symptoms, being unable to reverse the state of immune imbalance. However, not all the patients could get benefit from the partially pharmacotherapy-based relief of the symptoms. It was reported that pharmacotherapy could confer the partial or poor relief to the one-third of children and almost two-thirds of adults AR patients [12]. Although the specific immunotherapy can desensitize patients and prevent disease progression, its overwhelming shortcomings limit clinical applications, such as long treatment cycle, poor patient compliance, and lacks long-term observation of large sample efficacy. In addition, specific immunotherapy is allergen-specific instead of allergen versatile. Surgery is less applied due to its controversy. Thus, to cure the AR patients effectively and fundamentally, new therapeutic strategies are indispensable.

## 3. AR and MSCs

### 3.1. Immunomodulatory Properties of MSCs

It is well known that the MSCs lead to a shift from Th2 to Th1 responses in AR and can regulate the functions of regulatory T cells (Tregs) as well [13, 14]. Although the basic mechanisms of MSCs immunomodulation remain to be elusive, it is plausible to speculate that the immunomodulation conferred by the MSCs might be mediated by soluble factors and direct cell-to-cell contact. Indeed, the MSCs can target several subsets of lymphocytes, including CD4+ Th cells, CD8+ cytotoxic T-lymphocytes (CTLs), natural killer (NK) cells, NKT cells, B cells, DCs, and Tregs [15]. What is more, the MSCs regulate the adaptive and innate immune system by suppression of T cells and maturation of DCs, reducing the activation and proliferation of B cells, inhibiting the proliferation and cytotoxicity of NK cells and promoting the generation of Tregs by soluble factors or cell-cell contact mechanisms [16–18].

**Figure 1:**
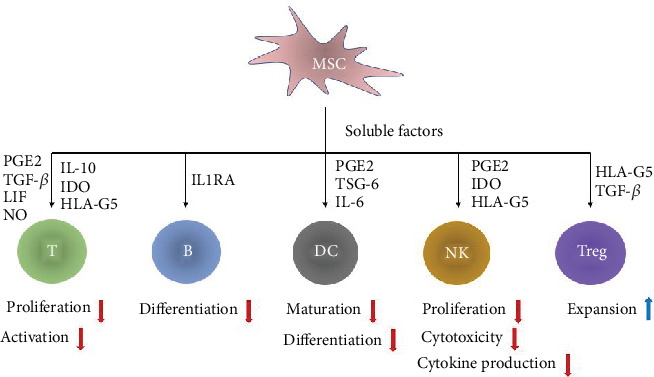
Schematic illustration of soluble factors for MSCs-mediated immunosuppression. MSCs exert their immunosuppression effects by secreting various soluble factors. MSCs inhibit the proliferation and activation of T cells, suppress B cell differentiation, inhibit the maturation and differentiation of DCs, suppress the proliferation, cytotoxicity, and cytokine production of NK cells. MSCs also induce Tregs expansion.

The capacity of MSCs that alter phenotype and function of immune cells largely attributes to the production of soluble factors. MSCs produce and release various soluble factors that are accountable for the immunosuppression function, including prostaglandin E2 (PGE2) [19–21], indoleamine 2,3-dioxygenase (IDO) [20–22], transforming growth factor-*β* (TGF-*β*) [21, 23], interleukin (IL)-10 [22, 24], nitric oxide (NO) [25], TNF-stimulated gene 6 (TSG-6) [26], IL-6 [27], leukemia inhibitory factor (LIF) [28], human leukocyte antigen (HLA)-G5 [14], and interleukin 1 receptor antagonist (IL1RA) [29] (Table 1). MSCs could interact with immune cells by secreting multiple soluble factors to exert immunosuppression effects (Figure 1).

Han et al. [30] found that MSCs suppressed the survival as well as the proliferation of T cells by mainly the contact-dependent mechanisms and resulted expansion of Tregs. Similarly, Fu et al. found that MSCs derived from human induced pluripotent stem cells (iPSCs) are capable of modulating T-cell phenotypes towards Th2 suppression through inducing Tregs expansion, which was associated with cell contact and PGE2 production [31]. Further, Dorronsoro et al. believed that Human MSCs modulated T-cell responses through TNF-*α*-mediated activation of nuclear factor kappa B (NF-*κ*B) [32].

In contrast to the suppressive activity on activated T cells, MSCs promoted the proliferation and activation of T cells in the quiescent state. Fan et al. reported that iPSC-MSCs balanced biased Th1/Th2 cytokine levels via promoting the proliferation of resting lymphocytes, activating CD4+ and CD8+ T cells, and upregulating Tregs without any additional stimulation. The further study demonstrated that cell-to-cell contact could be a mechanism possibly involved in the immunomodulation, while the NF-*κ*B was identified to play an important role in the immunomodulatory effects of iPSC-MSCs on quiescent T cells [33].

MSCs had immunosuppressive effect on activated T cells but could promote the responses of quiescent T cells, which suggested different immunomodulatory functions of MSCs according to the phases of diseases.

However, Desai et al. investigated the immune effects of MSCs on allergen-stimulated lymphocytes from AR subjects and found that in contrast to subjects with allergic asthma, MSCs caused a significant increase in the proliferation of antigen challenged lymphocytes from AR subjects. In their opinion, the increase in lymphocyte proliferation was caused by the MSCs presenting the allergens to CD4+ T cells, which was correlated with increased production of inflammatory cytokines from T cells, and increased expressions of major histocompatibility complex (MHC)-II and CD86 on MSCs [34]. These contradictory findings suggest that further research is needed to clarify the immunomodulatory function and mechanism of MSCs in AR.

### 3.2. Potential of the MSCs for AR Therapy

Currently, emerging evidences are addressing the potential of MSCs for immunomodulatory mechanism in an animal model of AR (Table 2) and indicated that different tissues derived MSCs functioned similar immunomodulatory effects.

#### 3.2.1. The Adipose- Derived MSCs

It was reported that in the mouse model of AR, adipose-derived MSC could migrate to the nasal mucosa and inhibit eosinophilic inflammation partially via shifting to a Th1 from a Th2 immune response to allergens [35]. Ebrahim et al. compared the immunomodulatory effects conferred by the adipose-derived MSCs versus montelukast, a leukotriene receptor antagonist, in the ovalbumin(OVA)-induced AR rat model. It was found that both the montelukast and the MSCs could significantly reduce allergic symptoms and the OVA-specific IgE, IgG1, IgG2a, and histamine accordingly, while increased PGE2. Furthermore, the significant suppression was observed in the induction of nasal innate cytokines, such as IL-4 and TNF-*α*, and chemokines, such as C-C Motif Chemokine Ligand 11 (CCL11) and vascular cell adhesion molecule-1(VCAM-1). However, the TGF-*β* induction was upregulated in both the MSCs and the montelukast groups with a more significant effect in the MSCs-treated group. More interestingly, the adipose tissue-derived MSCs-treated group demonstrated more restoring effects on the structure of the nasal mucosa [36].

#### 3.2.2. The Tonsil- Derived MSCs

The MSCs derived from human tonsil could effectively reduce allergic symptoms, Th2 cytokines, and OVA-specific IgE secretion from B cells in a mouse model of AR. Moreover, the levels of the innate cytokine (IL-25 and IL-33) and eotaxin mRNA were decreased in the nasal mucosa, suggesting this mechanism contributing to the reduced allergic inflammation [37].

#### 3.2.3. The Nasal Mucosa-Derived MSCs

Yang et al. reported that the nasal mucosa-derived MSCs from mice could migrate to nasal mucosa via tail vein injection in the OVA-sensitized mice. More importantly, these MSCs were proved to be regulators that balanced the Th1 and Th2 immune responses by upregulating IgG2a and interferon (IFN)-*γ* and downregulating IgE, IgG1, IL-4, IL-5, and IL-10 [38].

#### 3.2.4. The Bone Marrow-Derived MSCs

Zhao et al. demonstrated that intravenous injection of the bone marrow-derived MSCs in the mouse model of AR significantly alleviated allergic symptoms and reduced the eosinophil infiltration, OVA-specific IgE, Th2 cytokine profile (IL-4, IL-5, and IL-13), and regulatory cytokines (IL-10). Accordingly, the level of Th1 (IFN-*γ*) increased significantly after MSCs treatment [39]. A similar discovery was made in a separate study. It was found that bone marrow-derived MSCs migrated to the nasal and lung tissues following intraperitoneal delivery and ameliorated to the airway remodeling and airway inflammation both in the upper and lower airways via the inhibition of Th2 immune response in the mouse model of AR [40].

#### 3.2.5. The Umbilical Cord-Derived MSCs

Li et al. found that human umbilical cord-derived MSCs ameliorate acute AR in rats likely via its regulation of the related cytokines secretion from macrophages during the acute AR. The physiological evidences included the MSCs-conferred reduction of IL-4, TNF-*α*, and IgE levels in the serum, as well as the MSCs-mediated inhibition of histamine and the recruitment of macrophages in the nasal mucosa [41].

Although up to date, the MSCs-mediated effects on the AR therapy were observed in animal models only; it shed light on the promising future to come for the potential therapeutic applications in the MSCs-based AR treatments.

## 4. Perspectives

The studies on the MSCs-based therapy in AR animal models could provide an alternative and very promising strategy for more effectively and essentially benefiting the AR patients who cannot be cured with traditional therapies. However, it still has a long way to go from the current studies in the AR animal models to the final clinical application for the AR therapy safely, effectively, and routinely due to some big challenges we are facing as detailed below.

Technically, the current methods for the MSCs generation are lacking in efficiency and high quality. (1) It is unclear how to develop high-quality clinical-grade MSCs products. (2) Quality control for the MSCs generated so far is a big concern because the MSCS generated from the different tissues and by different labs were based on their own protocols. (3) Significant variations in preparation, adaptability, and functionality of the MSCs due to tissue sources, culture methods, and propagation levels [42] add more uncertainty to the study and the clinical application. (4) Although the MSCs-based therapy could confer the significant therapeutic effects on AR symptoms in animal models, the potential cellular changes during the generation of MSCs might occur and bring the unknown influences for the clinical therapy. (5) So far in almost all the cases, the MSCs are generated and propagated under in vitro conditions instead of the normal physiological in vivo conditions, possibly affecting the biological properties of the generated MSCs. More specifically, some potential risks in MSCs generation and propagation under the nonphysiological conditions, such as oxygen level, cell density, culture medium ingredient and quality, number of passages, and proliferative senescence. All these uncertainties may significantly alter the MSCs' quality and properties [43].

Biologically, it is essential to further investigate the mechanism of how the MSCs regulate the immunomodulation to cure the AR symptoms immunologically. Clinically, to make the translation happen safely, ethically, and effectively, it is indispensable to accumulate the clinical efficacy and long-term safety data. More specifically, for the clinical trials, the information on the MSCs dosage and application methods serves as the prerequisite for bringing the MSC-based therapy in AR animal models into the clinic.

Recent studies have revealed that extracellular vesicles (EVs) derived from MSCs (MSC-EVs) might carry similar immunomodulatory properties of MSCs [44, 45]. EVs are bilayer membrane structures carrying various biomolecules, such as RNAs and proteins. Compared with whole-cell therapy, MSC-EVs have significant advantages, such as low immunogenicity, high biosafety, and convenient storage. Therefore, MSC-EVs have been identified as novel and promising cell-free therapeutic agents. However, there are few studies on the treatment of AR with MSC-EVs. Fang et al. demonstrated that MSC-EVs were able to prevent allergic airway inflammation through the delivery of miR-146a-5p, suggesting that MSC-EVs could be a novel strategy for the treatment of AR [46]. A variety of further investigations are required to precisely elucidate the efficacy and underlying mechanisms of EVs-based therapy in AR.

## Figures and Tables

**Table 1:** Soluble factors critical for MSCs-mediated immunosuppression.

Soluble factors	Immunomodulatory effect	Reference
PGE2	Inhibiting the maturation of DCs Inhibiting the proliferation, cytotoxicity, and cytokine production of NK cells Suppressing CD8+ T cell-mediated activation	[19–21]
IDO	Inhibiting the proliferation, cytotoxicity, and cytokine production of NK cells Suppressing the proliferation of T cells Suppressing CD8+ T cell-mediated activation	[20–22]
TGF-*β*	Suppressing CD8+ T cell-mediated activation Inducing Tregs	[21, 23]
IL-10	Suppressing the proliferation of T cells Inhibiting Th17 cell differentiation	[22, 24]
NO	Suppressing the proliferation of T cells	[25]
TSG-6	Inhibiting the maturation and function of DCs	[26]
IL-6	Inhibiting the differentiation of DCs	[27]
LIF	Inhibiting the proliferation of T cells	[28]
HLA-G5	Suppressing the proliferation of T cells Inducing the expansion of Tregs Inhibiting the cytotoxicity and cytokine production of NK cells	[14]
IL1RA	Suppressing the differentiation of B cells	[29]

**Table 2:** Summary of the applications of MSCs in AR model.

Animals	Source of MSCs	Administration and dosage	Effect	Reference
BALB/c mice	BALB/c mice adipose tissue	Tail vein injection, 2 × 10^6^, once a day for 3 days	Y	[35]
Albino rats	Albino rats adipose tissue	Intraperitoneal injection, 1 × 10^6^, weekly for 3 weeks	Y	[36]
BALB/c mice	Human tonsil tissue	Intravenous injection, 0.5 × 10^6^, once a day for 6 days	Y	[37]
Mice	Mice nasal mucosa	Tail vein injection, once a day for 3 days	Y	[38]
BALB/c mice	BALB/c mice bone marrow	Intravenous injection, 0.5 × 10^6^, once a day for 2 weeks	Y	[39]
BALB/c mice	BALB/c mice bone marrow	Intraperitoneal injection, 1 × 10^6^/2 × 10^6^, 1 dose	Y	[40]
Sprague-Dawley rats	Human umbilical cord	Intraperitoneal injection, 5 × 10^6^/2 × 10^6^, 1 dose before/after AR rat model construction or weekly for 4 weeks after AR rat model construction	Y	[41]

Abbreviations: Y: effect was shown.
